# Lp(a), platelet count, aspirin and cardiovascular outcome: a prospective cohort analysis

**DOI:** 10.3389/fcvm.2026.1932150

**Published:** 2026-09-21

**Authors:** Xuan Song, Jia-Xin Huang, Qian Hou, Jing Chen, Hong Shu, Li-Chan Tao, Jian-Jun Li

**Affiliations:** 1 Department of Endocrinology, The Third Affiliated Hospital of Soochow University, Changzhou, China; 2 Department of Cardiology, The Third Affiliated Hospital of Soochow University, Changzhou, China; 3 Cardiometabolic Center, State Key Laboratory of Cardiovascular Diseases, Fu Wai Hospital, National Center for Cardiovascular Diseases, Chinese Academy of Medical Sciences and Peking Union Medical College, Beijing, China

**Keywords:** aspirin, cardiovascular risk, lipoprotein(a), platelet count, primary prevention

## Abstract

**Background:**

Elevated lipoprotein(a) [Lp(a)] and increased platelet count independently predict major adverse cardiovascular events (MACE), yet their interplay and joint value in cardiovascular risk stratification remain incompletely elucidated. We examined their separate and joint associations with incident MACE, with a secondary exploratory analysis of whether preventive benefit from aspirin differs across biomarker-defined subgroups.

**Methods and results:**

We included 250,927 participants free of prior cardiovascular disease (CVD) from the UK Biobank (enrolled 2006–2010). Participants were categorized into four groups based on Lp(a) status (elevated: >125 nmol/L) and platelet count (increased: upper tertile, >273 × 10^9^/L). Multivariable Cox regression was applied to estimate hazard ratios (HRs) for MACE, adjusted for demographic, lifestyle, metabolic and medication-related covariates. Formal tests for additive and multiplicative interactions were performed. Over a median follow-up of 13.4 years, both elevated Lp(a) (HR 1.250; 95% CI 1.118–1.325) and increased platelet count (HR 1.056; 95% CI 1.005–1.110) were independently associated with higher MACE risk. Participants with concurrent elevation of both biomarkers carried the highest MACE risk (HR 1.391; 95% CI 1.256–1.541). Neither additive nor multiplicative interaction reached statistical significance, indicating that the joint effect reflects additive risk accumulation rather than biological synergy. In exploratory subgroup analyses, aspirin use was associated with lower MACE risk only in the double-high subgroup, but not in those with isolated elevated Lp(a).

**Conclusion:**

Concomitant elevation of Lp(a) and platelet count identifies a subgroup of individuals at substantially higher risk for MACE. The observed potential benefit of aspirin in the double-high subgroup is exploratory and warrants further prospective investigation.

## Graphical Abstract

**Figure:**
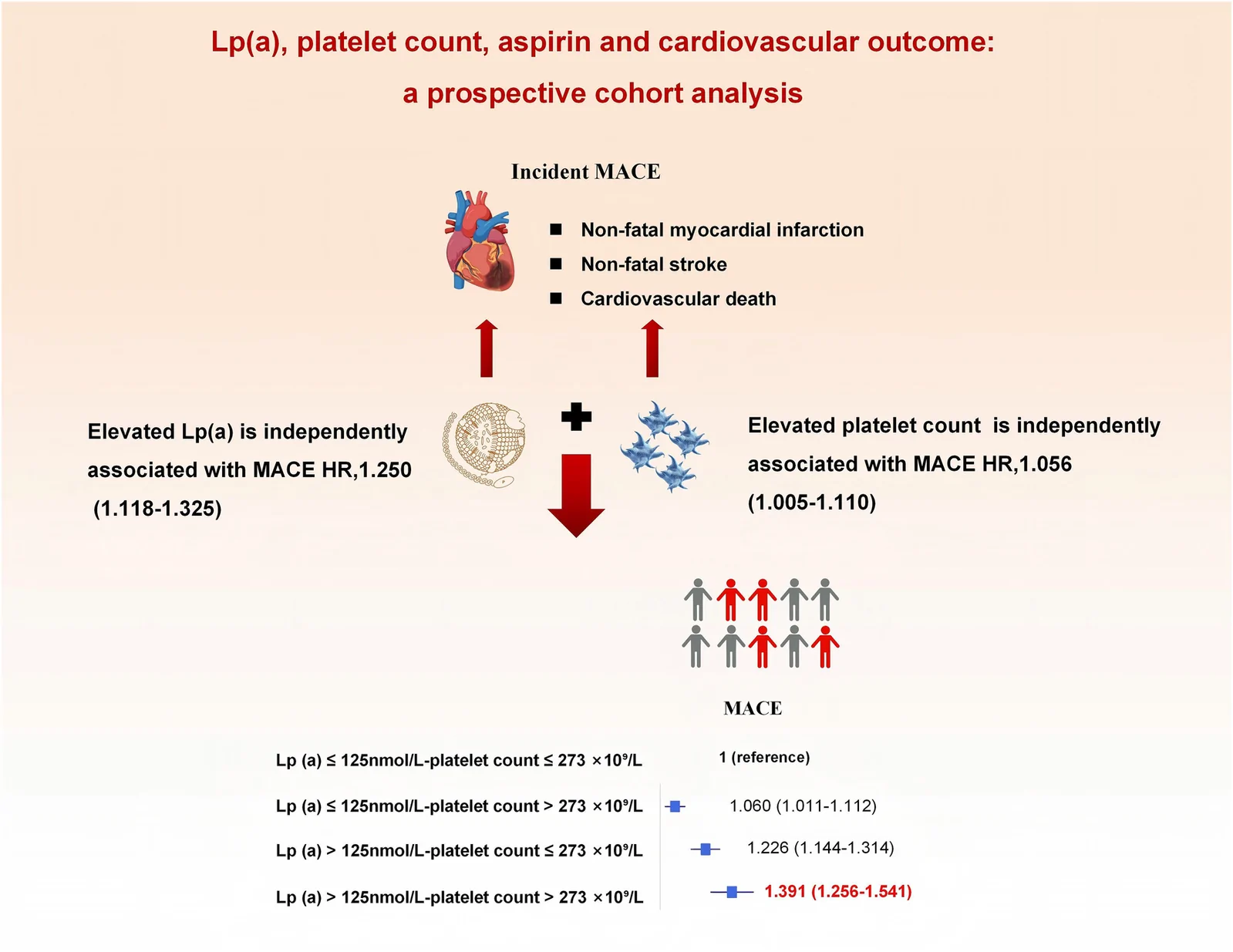
Schematic summary of the study design and main findings. Among 250,927 UK Biobank participants without prior cardiovascular disease, elevated Lp(a) (>125 nmol/L) and increased platelet count (>273×10⁹/L) were independently associated with incident MACE (composite of non-fatal myocardial infarction, non-fatal stroke, and cardiovascular death) over a median follow-up of 13.4 years. Compared with the double-low group (Lp(a) ≤125 nmol/L and platelet count ≤273×10⁹/L), participants with both biomarkers elevated showed the highest MACE risk, while those with isolated elevation of Lp(a) or platelet count had intermediate risks. Abbreviations: HR, Hazard ratio; Lp(a), Lipoprotein(a); MACE, major adverse cardiovascular events.

## Introduction

Cardiovascular disease (CVD) remains a leading cause of global morbidity and mortality, driving the relentless pursuit of refined risk stratification strategies. While established risk factors are instrumental, a significant residual risk persists, underscoring the critical need for novel biomarkers (1, 2). Among these, lipoprotein(a) [Lp(a)] has emerged as a pivotal, genetically determined risk marker. Large-scale epidemiological studies, including recent analyses from the UK Biobank (UKB) (3), have robustly confirmed that elevated Lp(a) levels, particularly concentrations exceeding 125 nmol/L, are independently associated with a significantly increased risk of atherosclerotic cardiovascular disease (4), peripheral arterial disease (5), and aortic valve stenosis (4, 6).

The robust association between Lp(a) and CVD is supported by multifaceted pathophysiological mechanisms. Beyond its established role in promoting atherogenesis through cholesterol deposition (7), Lp(a) exerts pro-inflammatory effects (8) and carries pro-atherogenic oxidized phospholipids (9). Particularly relevant to thrombotic risk is its structural homology with plasminogen, through which it inhibits fibrinolysis and enhances thrombogenesis (10–12). However, despite these clear mechanisms and its strong genetic determination, risk prediction based solely on Lp(a) levels remains imprecise. A critical knowledge gap exists in understanding how and in whom this inherent genetic risk translates into clinical events. The pathways through which Lp(a) ultimately confers CVD risk are likely modulated by a spectrum of factors, including inflammatory status and lifestyle influences (13–15). This highlights the importance of identifying modifiable clinical factors that may exacerbate or mitigate Lp(a)-associated risk. Given its potent pro-thrombotic properties, investigating the interaction between Lp(a) and key components of the hemostatic system, particularly platelets, represents a compelling and mechanistically plausible research direction.

In this context, platelet count emerges as a promising and clinically accessible candidate that may integrate the influence of modifiable pathways on thrombotic risk. Epidemiological studies have linked higher platelet counts, even within the normal range, to increased CVD risk (16, 17). An elevated platelet count can be a marker of enhanced thrombopoiesis and a pro-thrombotic state, which is often influenced by modifiable factors such as chronic low-grade inflammation related to diet, physical activity, and hyperlipidemia (18–20). This positions platelet count as a potential clinical bridge between genetically determined Lp(a) elevation and the modifiable inflammatory milieu that may modulate its risk. The biological plausibility for a potential interplay between Lp(a) and platelet count is supported by evidence that oxidized phospholipids (OxPLs), which are prominently carried by Lp(a) (9, 21), can enhance platelet activation and adhesion (11, 22). Thus, in individuals with elevated Lp(a), a higher platelet count—potentially driven by inflammation, may signify an additional pro-thrombotic burden these distinct mechanisms.

Nevertheless, it remains unclear whether platelet count clinically modifies the association between elevated Lp(a) and incident CVD. We therefore hypothesize that platelet count is an important clinical modifier of Lp(a)-associated cardiovascular risk, and that individuals with concomitant elevation of both Lp(a) and platelet count represent a distinct subgroup at significantly heightened risk for CVD events. To test this hypothesis, this study aims to systematically evaluate the interaction between Lp(a) and platelet count in predicting major adverse cardiovascular events (MACE) within the large, prospective UKB cohort. Furthermore, to preliminarily explore the biological plausibility of this interaction, we will assess whether the protective association of aspirin use differs within this high-risk subgroup.

## Methods

### Data source and study population

This study utilized data from the UKB, a large-scale database containing genetic, phenotypic, and health information from participants across the United Kingdom. Participants were recruited from 22 centers across the UK between 2006 and 2010. All participants completed a structured touch-screen questionnaire and underwent comprehensive baseline assessments, including detailed sociodemographic profiling, lifestyle evaluations, health status examinations, and physical assessments. Data linkage to primary care and hospital records provided details on hospital admissions and outcome events, while linkage to death registries offered comprehensive follow-up on mortality outcomes. Cases are defined according to the International Classification of Diseases, Tenth Revision (ICD-10) codes, ensuring standardized and consistent categorization of all health outcomes across the cohort.

A total of 502,301 participants were included during the baseline assessment from 2006 to 2010. First, we excluded 56,742 individuals with prevalent cardiovascular events at baseline. Subsequently, we excluded participants with missing data for the key exposure variables, Lp(a) and platelet count. Finally, we further excluded participants with missing data for other key covariates. The final analytical cohort comprised 250,927 participants. A detailed depiction of the participant selection process is provided in the study flowchart (Figure 1).

**Figure 1:**
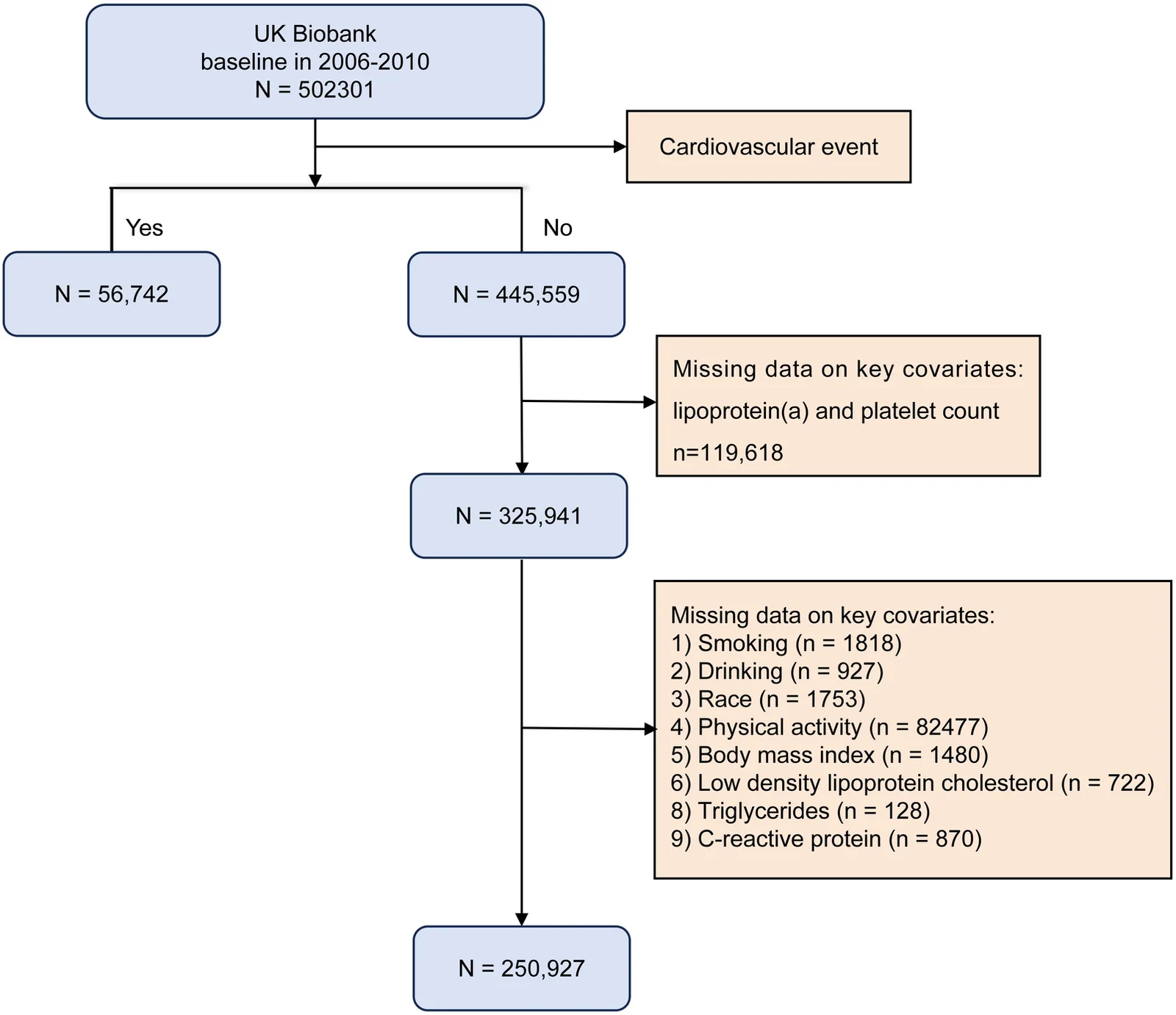
Flowchart of participant selection.

### Lipoprotein(a) assessment

Lp(a) levels were measured using standardized immunoturbidimetric immunoassays across UKB assessment centers. For the primary analysis, Lp(a) levels were categorized as ≤125 nmol/L and >125 nmol/L, based on established thresholds linked to increased cardiovascular risk. Measurements were conducted using the Randox Laboratories immunoturbidimetric assay on the Beckman Coulter AU5800 analyzer (Data Field 30790).

### Platelet count assessment

Platelet counts were quantified within 24 h of sample collection at the UKB central laboratory. Using Beckman Coulter LH750 hematology analyzers, measurements were performed on whole blood samples collected in EDTA vacuum containers. Given that tertiles are routinely used for stratification in clinical studies, we adopted the upper tertile as the threshold, which yielded two groups: lower (≤273 × 10^9^/L) and upper tertile (>273 × 10^9^/L) (23).

### Outcome measures

The primary outcome was the first occurrence of MACE, defined as a composite of non-fatal myocardial infarction (MI), non-fatal stroke, and cardiovascular death. Events were identified through linkage to hospital admission data and death registries using relevant ICD-10 codes (Supplementary Table 12). The last follow-up dates were October 31, 2022, for participants in England; July 31, 2021, for those in Scotland; and February 28, 2018, for those in Wales. During the follow-up period, participants experienced either a new outcome, death, or reached their last follow-up date, whichever occurred first.

### Demographics

Data on potential confounding variables were collected through structured questionnaires and interviews. These potential confounding variables included sociodemographic (age, gender, race, body mass index), lifestyle habits (current smoking status, current drinking status, physical activity), dietary scores, and medical history. Laboratory measurements comprised total cholesterol, high-density lipoprotein cholesterol (HDL-C), low-density lipoprotein cholesterol (LDL-C), triglycerides and C-reactive protein. Body mass index (BMI) was calculated from measured weight and height (kg/m^2^). Definitions of MET and diet score are provided in the supplementary methods. Anti-hypertensive and lipid-lowering medications were defined based on code 6,177 and 6,153. Disease diagnoses are detailed in Supplementary Table 12, and definitions of other medication use are presented in Supplementary Tables 13, 14.

### Statistical analysis

Participants' baseline profiles were illustrated by jointly assessing Lp(a) (using a cutoff of 125 nmol/L) and platelet count (using the upper tertile cutoff of 273 × 10^9^/L).Comparisons were made across four groups: Group 1 [Lp(a) ≤ 125 nmol/L & PLT ≤ 273 × 10^9^/L]; Group 2 [Lp(a) ≤ 125 nmol/L & PLT > 273 × 10^9^/L]; Group 3 [Lp(a) > 125 nmol/L & PLT ≤ 273 × 10^9^/L]; and Group 4 [Lp(a) > 125 nmol/L & PLT > 273 × 10^9^/L]. Normality assessment for continuous variables revealed that most distributions were skewed; therefore, all continuous variables are presented as median (first quartile, third quartile). Categorical variables are described as frequency (percentage). To evaluate the selection bias potentially introduced by the exclusion of participants with missing data for the key variables [Lp(a) and platelet count], baseline characteristics were compared between the final analysis cohort and the missing-data subgroup, and a supplementary analysis employing multiple imputation was additionally conducted to handle missing covariates.

We initially established cox regression models to independently assess the associations of Lp(a) and platelet count levels with MACE. We constructed three models: Model 1 accounted for age (years), gender, current smoking status, current drinking status, diet score, physical activity and BMI, while model 2 extended adjustments to include additional covariates: LDL-c, TG and CRP) Model 3 with additional adjustments for diabetes, hypertension, atrial fibrillation, chronic kidney disease, use of anti-hypertensive, hypoglycemic agent, lipid-lowering drugs, and other anti-platelet medications. The proportional hazards assumption of the Cox regression model was assessed using the Schoenfeld residual test, and no violation was observed (*P* > 0.05). Subsequently, a cox proportional hazards regression model was used to assess the risk of MACE across the four groups defined by combined Lp(a) and platelet count levels. Additionally, we quantified additive interaction between Lp(a) and platelet count on MACE risk. To explore the heterogeneity of the main associations across different populations, we conducted the following analyses: First, we performed stratification based on several predefined factors, including age, sex, and smoking history, to evaluate the consistency of the association between the Lp(a)-platelet count joint groups and MACE risk. Given that age is a key influencing factor, we separately assessed the association between the joint groups and MACE risk within the middle-aged (45–65 years) and older (>65 years) age strata. To explore potential heterogeneity in risks across different types of MACE, we performed subgroup analyses for the composite endpoint of non-fatal myocardial infarction, non-fatal stroke, and cardiovascular death. To further assess whether cardiovascular death risk was influenced by the competing risk of non-cardiovascular death, we employed Fine-Gray competing risks models, with cardiovascular death set as the primary event and non-cardiovascular death as the competing event, to calculate sub distribution hazard ratios for the joint Lp(a) and platelet count exposure groups. All covariates adjusted for in the multivariable Cox models were similarly adjusted in the competing risks models to ensure comparability of the results.

Given that aspirin is a key anti-platelet agent, we systematically evaluated its impact on the primary associations: First, assess the association between combined Lp(a) and platelet count groups and MACE risk, stratified by aspirin use. Furthermore, we incorporated product terms between aspirin use and Lp(a) as well as platelet count into the Cox model to test for multiplicative interaction. Furthermore, we stratified the population based on baseline aspirin use and, within each stratum, assessed: a) the independent risks associated with Lp(a) and platelet count; and b) the association between Lp(a) and MACE within subgroups defined by different platelet count levels (≤273 × 10^9^/L and >273 × 10^9^/L). Finally, to examine the robustness of the clinical cut-point for Lp(a) (125 nmol/L) used in the primary analysis and to explore potential dose-response relationships, we employed restricted cubic splines models. To further quantify this association and test the robustness of the main findings under different risk thresholds, we redefined risk strata based on the inflection points observed in the RCS curves and the 75th percentile of Lp(a) levels. We then evaluated the association between these redefined strata and MACE risk in the overall population and in populations stratified by aspirin use status. We further applied inverse probability of treatment weighting (IPTW) to balance measured covariates between aspirin users and non-users, and re-evaluated the association of the combined Lp(a) and platelet count groups with the risk of MACE. Given that the weight distribution fell within an acceptable range, no truncation of extreme weights was performed. Covariate balance between aspirin users and non-users was evaluated using standardized mean differences (SMD), with an SMD below 0.1 considered indicative of adequate balance.

All statistical analyses were conducted using R software (version 4.4.1) and SPSS Statistics (version 25.0; IBM Corp). All tests were two-sided, and a *P* value <0.05 was considered statistically significant.

## Results

### Population characteristics

This study included 250,927 participants from the UK Biobank (Table 1). The median follow-up duration was 13.4 years. Participants were divided into four groups based on Lp(a) levels (≤125 or >125 nmol/L) and platelet count (≤273 or >273 × 10^9^/L). Baseline characteristics showed significant differences in several cardiovascular risk factors across the groups, from the double-low group to the double-high group. In addition to the expected differences based on grouping variables, individuals in the double-high group tended to have higher levels of TC, LDL-C, and CRP. Significant differences were also observed in sex distribution, education level, household income, and the use of anti-hypertensive and lipid-lowering medications across the groups.

**Table 1:** Baseline characteristics of the study participants.

Characteristics	Group1 (*n* = 150,900)	Group2 (*n* = 72,670)	Group3 (*n* = 18,428)	Group4 (*n* = 8,929)	*P*-value
Age (years)	57.00 (49.00–63.00)	56.00 (48.00–62.00)	57.00 (50.00–63.00)	56.00 (49.00–62.00)	0.007
Male	79,669 (52.80%)	23,344 (32.10%)	9,364 (50.80%)	2,745 (30.70%)	<0.001
Ethnicity					<0.001
White	136,666 (90.60%)	66,068 (90.90%)	16,859 (91.50%)	8,237 (92.20%)	
Smoking history					0.029
Yes	89,517 (59.30%)	42,577 (58.60%)	10,930 (59.30%)	5,226 (58.50%)	
No	61,383 (40.70%)	30,093 (41.40%)	7,498 (40.70%)	3,703 (41.50%)	
Drinking history					0.002
Yes	145,606 (96.50%)	69,647 (95.80%)	17,381 (96.80%)	8,581 (96.10%)	
No	5,294 (3.50%)	3,023 (4.20%)	597 (3.20%)	348 (3.90%)	
Education	8.57 (3.34–18.65)	9.52 (3.75–20.44)	8.77 (3.42–19.03)	9.51 (3.81–20.57)	<0.001
Household income (£)					<0.001
<18 000	24,258 (17.90%)	12,804 (19.80%)	3,103 (18.70%)	1,595 (20.00%)	
18 000–30 999	32,548 (24.00%)	15,955 (24.70%)	4,005 (24.20%)	2,037 (25.60%)	
31 000–51 999	37,148 (27.40%)	17,808 (27.50%)	4,548 (27.50%)	2,127 (26.70%)	
52 000–100 000	32,056 (23.70%)	14,397 (22.30%)	3,861 (23.30%)	1,776 (22.30%)	
>100 000	9,443 (7.00%)	3,677 (5.70%)	1,039 (6.30%)	427 (5.40%)	
Physical activity					0.098
Low intensity	25,804 (17.10%)	13,925 (19.20%)	3,054 (16.60%)	1,729 (19.40%)	
Moderate intensity	77,405 (51.30%)	36,773 (50.60%)	9,433 (51.20%)	4,489 (50.30%)	
High intensity	47,691 (31.60%)	21,972 (30.20%)	5,941 (32.20%)	2,711 (30.30%)	
BMI (kg/m^2^)	26.34 (23.86–29.24)	26.48 (23.91–29.68)	26.35 (23.98–29.22)	26.56 (24.12–29.68)	<0.001
SBP (mmHg)	135.00 (123.50–148.00)	135.00 (123.50–148.00)	135.50 (124.00–148.50)	135.00 (124.00–148.00)	0.015
Height (cm)	170.00 (163.00–177.00)	166.00 (160.00–172.00)	170.00 (163.00–177.00)	165.00 (160.00–172.00)	<0.001
Weight (kg)	76.90 (66.80–87.80)	73.70 (64.60–84.60)	76.70 (67.00–87.10)	73.70 (65.00–84.50)	<0.001
TC (mmol/L)	5.61 (4.91–6.34)	5.80 (5.10–6.55)	5.83 (5.13–6.56)	5.98 (5.29–6.72)	<0.001
TG (mmol/L)	1.44 (1.01–2.10)	1.49 (1.06–2.14)	1.39 (0.99–2.00)	1.44 (1.04–2.08)	0.003
HDL-C (mmol/L)	1.40 (1.17–1.67)	1.44 (1.21–1.71)	1.43 (1.21–1.71)	1.46 (1.24–1.73)	<0.001
LDL-C (mmol/L)	3.49 (2.95–4.06)	3.61 (3.07–4.20)	3.65 (3.13–4.23)	3.77 (3.23–4.33)	<0.001
CRP (mg/L)	1.11 (0.57–2.24)	1.49 (0.72–3.16)	1.14 (0.58–2.30)	1.54 (0.77–3.26)	<0.001
Lp(a) (nmol/L)	16.67 (8.66–41.84)	17.20 (8.80–41.75)	153.80 (138.79–171.20)	155.18 (140.10–172.51)	<0.001
Platelet count (×10^9^/L)	226.00 (200.00–248.80)	306.00 (287.70–334.10)	227.60 (201.20–249.60)	305.40 (287.15–333.70)	<0.001
Hypertension	23,397 (15.50%)	11,912 (16.40%)	2,852 (15.50%)	1,471 (16.50%)	<0.001
Diabetes	6,187 (4.10%)	3,094 (4.30%)	659 (3.60%)	375 (4.20%)	0.391
Atrial fibrillation	1,322 (0.90%)	518 (0.70%)	149 (0.80%)	65 (0.70%)	0.003
Chronic kidney disease	3,229 (2.10%)	1,493 (2.10%)	422 (2.30%)	190 (2.10%)	0.868
Use of anti-hypertensive	14,651 (9.70%)	4,413 (6.10%)	1,728 (9.40%)	520 (5.80%)	<0.001
Hypoglycemic agent	3,871 (2.60%)	1,930 (2.70%)	427 (2.30%)	228 (2.60%)	0.527
Lipid-lowering drugs	12,668 (8.40%)	3,325 (4.60%)	1,790 (9.70%)	439 (4.90%)	<0.001
Anti-platelet medications	15,230 (10.10%)	6,911 (9.50%)	1,928 (10.50%)	881 (9.90%)	0.219
Diet score					<0.001
0	1,915 (1.30%)	839 (1.20%)	237 (1.30%)	117 (1.30%)	
1	14,437 (9.60%)	6,606 (9.10%)	1,720 (9.30%)	777 (8.70%)	
2	34,997 (23.20%)	16,646 (22.90%)	4,219 (22.90%)	2,026 (22.70%)	
3	45,638 (30.20%)	22,217 (30.60%)	5,503 (29.90%)	2,662 (29.80%)	
4	37,646 (24.90%)	18,663 (25.60%)	4,639 (25.20%)	2,331 (26.10%)	
5	16,267 (10.80%)	7,699 (10.60%)	2,110 (11.40%)	1,016 (11.40%)	

Group 1: Lp(a) ≤ 125 nmol/L & platelet count ≤ 273 × 10^9^/L; Group 2: Lp(a) ≤ 125 nmol/L & platelet count > 273 × 10^9^/L; Group 3: Lp(a) > 125 nmol/L& platelet count ≤ 273 × 10^9^/L; Group 4: Lp(a) > 125 nmol/L& platelet count > 273 × 10^9^/L. The baseline characteristics of the study participants were divided into four groups (Group1-Group4). Continuous variables are presented as median (interquartile range), and categorical variables are described as frequency (percentage). The Jonckheere–Terpstra trend test was used for continuous variables; the Cochran–Armitage trend test was used for binary and ordered categorical variables; and Pearson's chi-square test was used for unordered categorical variables.

Physical activity levels were quantified using metabolic equivalent (MET) hours per week and categorized as: low intensity (< 10 MET-hours/week), moderate intensity (10–50 MET-hours/week), and high intensity (> 50 MET-hours/week). Diet score was derived from the following dietary factors: vegetable intake ≥4 tablespoons/day, fruit intake ≥ 3pieces/day, ﬁsh intake ≥ twice a week, unprocessed red meat intake ≤ twice a week, and processed meat intake ≤ twice a week. Each benefical dietary factor received one point, with the healthy eating score ranging from 0 to 5.

BMI, Body Mass Index; CRP, C-reactive protein; DBP, Diastolic blood pressure; HDL-C, High density lipoprotein cholesterol; LDL-C, Low density lipoprotein cholesterol; Lp(a), Lipoprotein(a); SBP, Systolic blood pressure; TC, Total cholesterol; TG, Triglycerides.

To evaluate potential selection bias from excluding participants with missing data for key variables [Lp(a) and platelet count], we compared baseline characteristics between the final analysis cohort and the subgroup with missing data (Supplementary Table 1). The subgroup with missing data showed higher risk profiles across several parameters, including age, cardiovascular risk factors (such as blood pressure, body mass index, diabetes, and chronic kidney disease), and the use of glucose-lowering and lipid-lowering medications.

### Individual association between platelet count, Lp(a) and MACE

Elevated Lp(a) was an independent risk factor for MACE (Table 2, HR = 1.250, 95% CI: 1.118–1.325), while platelet count was weakly associated with risk only in the highest tertile group (>273 × 10^9^/L) (Table 3, HR = 1.056, 95% CI: 1.005–1.110). To explore the continuous nature of the risk associations and validate the robustness of the categorical analysis, we performed two restricted cubic spline (RCS) analyses. Supplementary Figures 1 and 2 illustrate the continuous associations: log-transformed Lp(a) showed a predominantly positive monotonic association with MACE risk (with a significant non-linear component, *P* < 0.001), while platelet count exhibited a significant non-linear association (*P* < 0.001).

**Table 2:** Cox proportional hazards model assessing the impact of Lp(a) levels on MACE risk.

		HR (95% CI)		
Lp(a) level	Events/participants	Model 1	Model 2	Model 3
Lp(a) ≤ 125 nmol/L	8,748/223,570	1 (reference)	1 (reference)	1 (reference)
Lp(a) > 125 nmol/L	1,319/27,357	1.270 (1.199–1.346)	1.270 (1.199–1.346)	1.250 (1.118–1.325)

Values are HR (95% CI) derived from Cox regression models.

Model 1 was adjusted for age, sex, smoke, drink, ethnicity, diet score, physical activity and BMI. Model 2 was adjusted as Model 1 plus LDL-c, TG and CRP. Model 3 was adjusted as Model 2 plus diabetes, hypertension, atrial fibrillation, chronic kidney disease, use of anti-hypertensive, hypoglycemic agent, lipid-lowering drugs, and other anti-platelet medications.

BMI, Body Mass Index; CI, Confidence interval; CRP, C-reactive protein; HR, Hazard ratio; LDL-C, Low density lipoprotein cholesterol; Lp(a), Lipoprotein(a); TG, Triglycerides.

**Table 3:** The effect of categorizing platelet count into tertiles on the incident risk of MACE among participants.

		HR (95% CI)		
PLT level	Events/participants	Model 1	Model 2	Model 3
Q1	3,826/83,624	1 (reference)	1 (reference)	1 (reference)
Q2	3,285/83,554	1.015 (0.969–1.064)	1.001 (0.956–1.049)	0.991 (0.946–1.039)
Q3	2,956/83,749	1.094 (1.041–1.150)	1.064 (1.013–1.119)	1.056 (1.005–1.110)

Q1: platelet count < 227 × 10^9^/L, Q2: 227 × 10^9^/L ≤ platelet count ≤ 273 × 10^9^/L, Q3: platelet count > 273 × 10^9^/L; Values are HR (95% CI) derived from Cox regression models.

Model 1 was adjusted for age, sex, smoke, drink, ethnicity, diet score, physical activity and BMI. Model 2 was adjusted as Model 1 plus LDL-c, TG and CRP. Model 3 was adjusted as Model 2 plus diabetes, hypertension, atrial fibrillation, chronic kidney disease, use of anti-hypertensive, hypoglycemic agent, lipid-lowering drugs, and other anti-platelet medications.

BMI, Body Mass Index; CI, Confidence interval; CRP, C-reactive protein; HR, Hazard ratio; LDL-C, Low density lipoprotein cholesterol; Lp(a), Lipoprotein(a); PLT, Platelet Count; TG, Triglycerides.

### Combined impact of Lp(a) levels and platelet count on MACE risk

Participants were divided into four groups based on Lp(a) level and platelet count. The results showed a significant joint effect of elevated Lp(a) and increased platelet count on MACE risk. Compared with the double-low group, the double-high group [Lp(a) > 125 nmol/L and platelet count >273 × 10^9^/L] had the highest risk of MACE (HR = 1.391, 95% CI: 1.256–1.541) (Table 4). To assess the influence of missing covariate data, we performed a sensitivity analysis using multiple imputation for missing covariates among participants with complete Lp(a) and platelet count data (Supplementary Table 2), and we additionally applied inverse probability of treatment weighting to balance measured covariates between aspirin users and non-users (Supplementary Table 3). Following IPTW weighting, all covariates achieved satisfactory balance between aspirin users and non-users (Supplementary Table 4). In the imputed dataset, the overall pattern of associations across the four combined biomarker groups was directionally consistent with the primary analysis, providing complementary support for our main findings. However, given that this imputation was performed on a broader population than the complete-case cohort, these results should be interpreted as exploratory rather than confirmatory. Subgroup analyses showed that the effect of the Lp(a)-platelet count combination on cardiovascular risk was generally consistent across subgroups, but the results were not statistically significant in the non-drinking subgroup (Figure 2). After stratification by age, the joint effect of Lp(a) and platelet count on MACE risk remained significant. In the 45–65 years and >65 years age groups, compared with the double-low group [Lp(a) ≤ 125 nmol/L and platelet count ≤273 × 10^9^/L], the double-high group had a significantly increased risk of MACE, with HRs and 95% CIs of 1.264 (1.120–1.427) and 1.657 (1.336–2.055), respectively (Supplementary Table 5). We further quantified additive interaction (Table 5) between Lp(a) and platelet count using the relative excess risk due to interaction (RERI), attributable proportion (AP), and synergy index (SI). The RERI was 0.106 (95% CI: −0.059 to 0.270), the AP was 0.076 (95% CI: −0.037 to 0.189), and the SI was 1.369 (95% CI: 1.197–1.566), none of which reached statistical significance, indicating no evidence of additive synergy between the two markers (Table 5).

**Table 4:** Cox proportional hazards model assessing the combined impact of Lp(a) levels and platelet count on MACE risk.

		HR (95% CI)		
Lp(a) level and platelet count	Events/participants	Model 1	Model 2	Model 3
Lp(a) ≤ 125 nmol/L-platelet count ≤ 273 × 10^9^/L	6,255/150,900	1 (reference)	1 (reference)	1 (reference)
Lp(a) ≤ 125 nmol/L-platelet count > 273 × 10^9^/L	2,493/72,670	1.087 (1.036–1.139)	1.065 (1.015–1.116)	1.060 (1.011–1.112)
Lp(a) > 125 nmol/L-platelet count ≤ 273 × 10^9^/L	921/18,428	1.249 (1.166–1.339)	1.251 (1.167–1.340)	1.226 (1.144–1.314)
Lp(a) > 125 nmol/L-platelet count > 273 × 10^9^/L	398/8,929	1.437 (1.297–1.591)	1.404 (1.268–1.555)	1.391 (1.256–1.541)

Values are HR (95% CI) derived from Cox regression models.

Model 1 was adjusted for age, sex, smoke, drink, ethnicity, diet score, physical activity and BMI. Model 2 was adjusted as Model 1 plus LDL-c, TG and CRP. Model 3 was adjusted as Model 2 plus diabetes, hypertension, atrial fibrillation, chronic kidney disease, use of anti-hypertensive, hypoglycemic agent, lipid-lowering drugs, and other anti-platelet medications.

BMI, Body Mass Index; CI, Confidence interval; CRP, C-reactive protein; HR, Hazard ratio; LDL-C, Low density lipoprotein cholesterol; Lp(a), Lipoprotein(a); TG, Triglycerides.

**Figure 2:**
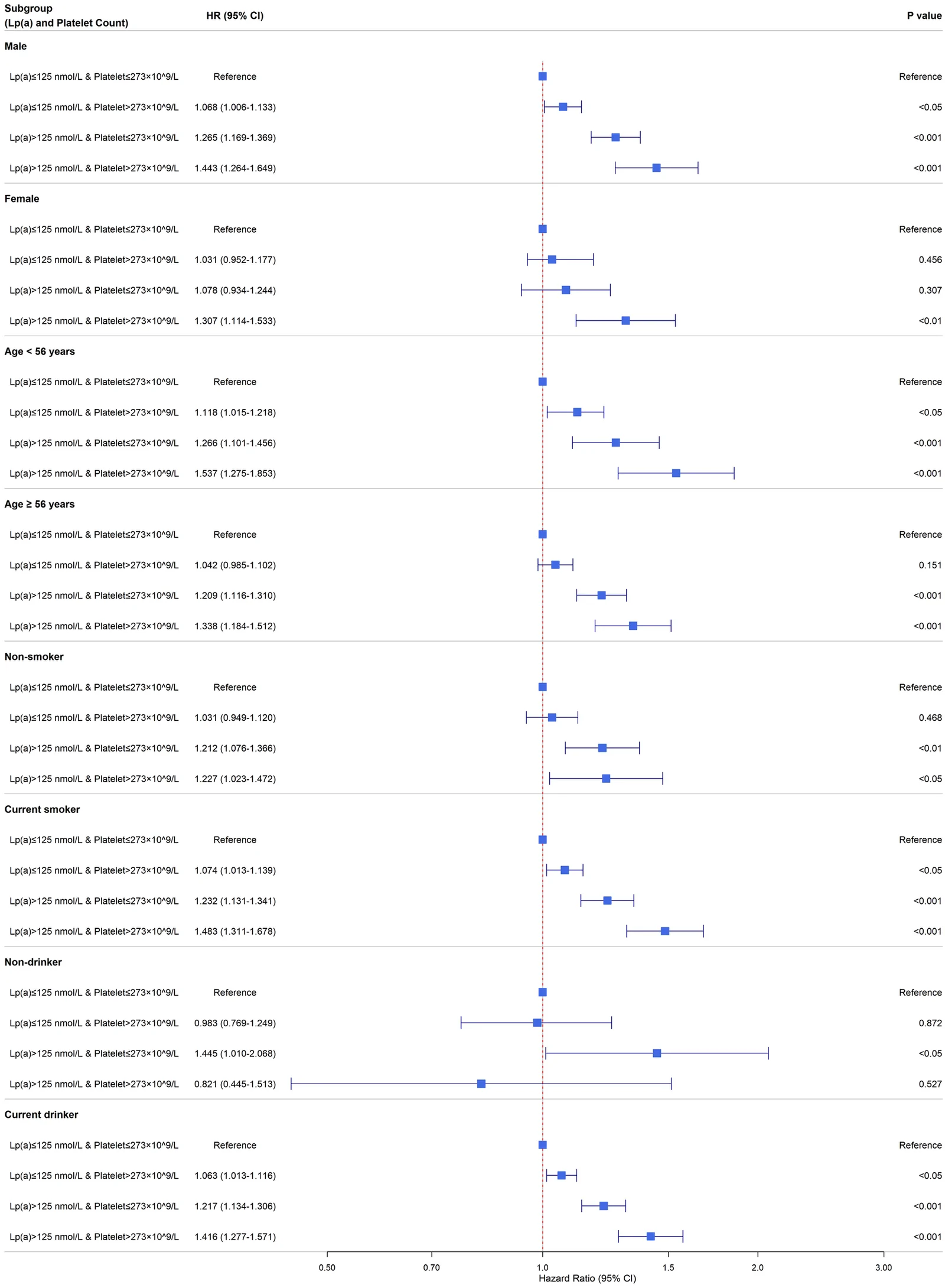
Subgroup analysis of the Cox proportional hazards model for combined Lp(a) and platelet count levels on MACE risk. Forest plot of multivariable-adjusted hazard ratios (HRs) for MACE according to combined Lp(a) (≤125 vs. > 125 nmol/L) and platelet count (≤273 vs. > 273 × 10^9^/L), with the low–low group as reference. Adjusted for all covariates detailed in Methods. BMI, Body Mass Index; CI, Confidence interval; CRP, C-reactive protein; HR, Hazard ratio; LDL-C, Low density lipoprotein cholesterol; Lp(a), Lipoprotein(a); TG, Triglycerides.

**Table 5:** Assessment of the additive interaction between Lp(a) and platelet count on incident MACE among the participants.

Lp(a) level and platelet count	RERI (95% CI)	AP (95% CI)	SI (95% CI)
Lp(a) ≤ 125 nmol/L-platelet count ≤ 273 × 10^9^/L	0.106 (−0.059–0.270)	0.076 (−0.037–0.189)	1.369 (1.197–1.566)
Lp(a) ≤ 125 nmol/L-platelet count > 273 × 10^9^/L			
Lp(a) > 125 nmol/L-platelet count ≤ 273 × 10^9^/L			
Lp(a) > 125 nmol/L-platelet count > 273 × 10^9^/L			

Values are HR (95% CI) derived from Cox regression models.

Model was adjusted for age, sex, smoke, drink, ethnicity, diet score, physical activity, BMI, LDL-c, TG, CRP, diabetes, hypertension, atrial fibrillation, chronic kidney disease, use of anti-hypertensive, hypoglycemic agent, lipid-lowering drugs, and other anti-platelet medications. BMI, Body Mass Index; CI, Confidence interval; CRP, C-reactive protein; HR, Hazard ratio; LDL-C, Low density lipoprotein cholesterol; Lp(a), Lipoprotein(a); TG, Triglycerides.

In Table 6, the stratified analyses based on MACE endpoints showed distinct risk profiles. The risk of non-fatal myocardial infarction showed the strongest association with the combined exposure status (HR = 1.371, 95% CI: 1.212–1.551). The risk of non-fatal stroke was elevated in the increased platelet count group and the double-high group, whereas the risk of cardiovascular death was primarily driven by elevated Lp(a). We additionally performed a competing risk analysis. In the Fine-Gray model treating non-cardiovascular death as a competing event, the double-high group [Lp(a) > 125 nmol/L and platelet count >273 × 10^9^/L] remained significantly associated with the risk of cardiovascular death (sHR = 1.340, 95% CI: 1.021–1.714), indicating that the combined exposure was still associated with an increased risk of cardiovascular death after accounting for competing risks (Supplementary Table 6).

**Table 6:** Cox proportional hazards model assessing the combined impact of Lp (a) levels and platelet count on non-fatal myocardial infarction, non-fatal stroke and cardiovascular death among the participants.

	Non-fatal myocardial infarction-HR (95% CI)		
Lp(a) level and platelet count	Model 1	Model 2	Model 3
Lp(a) ≤ 125 nmol/L-platelet count ≤ 273 × 10^9^/L	1 (reference)	1 (reference)	1 (reference)
Lp(a) ≤ 125 nmol/L-platelet count > 273 × 10^9^/L	1.021 (0.964–1.082)	1.004 (0.947–1.064)	1.000 (0.944–1.060)
Lp(a) > 125 nmol/L-platelet count ≤ 273 × 10^9^/L	1.281 (1.180–1.390)	1.283 (1.183–1.393)	1.253 (1.155–1.360)
Lp(a) > 125 nmol/L-platelet count > 273 × 10^9^/L	1.411 (1.248–1.596)	1.385 (1.224–1.566)	1.371 (1.212–1.551)

	Non-fatal stroke-HR (95% CI)		
	Model 1	Model 2	Model 3
Lp(a) ≤ 125 nmol/L-platelet count ≤ 273 × 10^9^/L	1 (reference)	1 (reference)	1 (reference)
Lp(a) ≤ 125 nmol/L-platelet count > 273 × 10^9^/L	1.130 (1.017–1.255)	1.120 (1.007–1.245)	1.124 (1.011–1.250)
Lp(a) > 125 nmol/L-platelet count ≤ 273 × 10^9^/L	1.146 (0.973–1.350)	1.132 (0.961–1.333)	1.127 (0.956–1.328)
Lp(a) > 125 nmol/L-platelet count > 273 × 10^9^/L	1.340 (1.061–1.691)	1.311 (1.038–1.656)	1.317 (1.143–1.664)

	Cardiovascular death-HR (95% CI)		
	Model 1	Model 2	Model 3
Lp(a) ≤ 125 nmol/L-platelet count ≤ 273 × 10^9^/L	1 (reference)	1 (reference)	1 (reference)
Lp(a) ≤ 125 nmol/L-platelet count > 273 × 10^9^/L	1.098 (0.993–1.215)	1.080 (0.976–1.195)	1.067 (0.948–1.200)
Lp(a) > 125 nmol/L-platelet count ≤ 273 × 10^9^/L	1.239 (1.062–1.445)	1.233 (1.057–1.439)	1.203 (1.007–1.436)
Lp(a) > 125 nmol/L-platelet count > 273 × 10^9^/L	1.301 (1.036–1.634)	1.271 (1.011–1.598)	1.320 (1.017–1.712)

Values are HR (95% CI) derived from Cox regression models.

Model 1 was adjusted for age, sex, smoke, drink, ethnicity, diet score, physical activity and BMI. Model 2 was adjusted as Model 1 plus LDL-c, TG and CRP. Model 3 was adjusted as Model 2 plus diabetes, hypertension, atrial fibrillation, chronic kidney disease, use of anti-hypertensive, hypoglycemic agent, lipid-lowering drugs, and other anti-platelet medications.

BMI, Body Mass Index; CI, Confidence interval; CRP, C-reactive protein; HR, Hazard ratio; LDL-C, Low density lipoprotein cholesterol; Lp(a), Lipoprotein(a); TG, Triglycerides.

### Aspirin on MACE risk associated with the combination of Lp(a) levels and platelet count

Further stratified analysis by aspirin use was performed to investigate the modifying effect of aspirin on the association between the combined groups and MACE risk. The combined risk of high Lp(a) and increased platelet count persisted among individuals not taking aspirin (HR = 1.441, 95% CI: 1.287–1.615). However, in the subgroup taking aspirin, the increased risk in the double-high group was no longer statistically significant, suggesting that aspirin was associated with a partial attenuation of the joint risk associated with high Lp(a) and increased platelet count (Supplementary Table 7). Further subgroup analysis (Supplementary Table 8) revealed an important modifying effect. Among individuals not taking aspirin, both increased platelet count and high Lp(a) were risk factors. However, in the subgroup taking aspirin, the association for increased platelet count alone was no longer significant (HR = 0.929, 95% CI: 0.840–1.028). In contrast, the risk associated with high Lp(a) remained elevated in the increased platelet count subgroup (HR = 1.310, 95% CI: 1.022–1.679). This suggests that aspirin may be mainly associated with attenuation of the risk pathway mediated by increased platelet count, but does not offset the residual risk associated with elevated Lp(a). We further evaluated multiplicative interaction by including product terms (Supplementary Table 9) and results showed that a significant interaction was observed between aspirin use and platelet count (HR for interaction = 0.874, *P* = 0.016), indicating that the association between aspirin use and lower MACE risk was more pronounced among individuals with higher platelet counts. In contrast, no significant interaction was detected between aspirin use and Lp(a) (*P* for interaction = 0.923).

The joint effect of elevated Lp(a) and platelet count on MACE risk, as well as its potential modification by aspirin use, remained robust when using an alternative Lp(a) cutoff (75th percentile) combined with the same platelet threshold, as well as when using data-driven cutoffs from RCS models (Supplementary Tables 10, 11).

## Discussion

In the present study based on the UKB dataset, we observed that concomitant elevations in Lp(a) (>125 nmol/L) and platelet count (>273 × 10^9^/L) were jointly associated with increased risk for MACEs. In exploratory subgroup analyses, aspirin appeared to be associated with reduced MACE risk among participants with combined elevation of both biomarkers, whereas no such association was observed in those with isolated elevated Lp(a).

We found both elevated Lp(a) and increased platelet count to be independent risk factors for MACE. The lack of statistically significant multiplicative or additive interactions supports an “effect accumulation” model, wherein the two markers operate through predominantly distinct biological pathways that summate their individual risk contributions. This indicates that, in our study population, Lp(a) and platelet count likely operate through predominantly distinct biological pathways that converge to increase overall risk.

Although based on observational data, this model of parallel risk contribution aligns well with the established multifactorial pathogenicity of Lp(a) and the distinct role of platelets. Lp(a) promotes cardiovascular risk through pathways that are not primarily dependent on platelet activity: it drives atherogenesis and carries pro-inflammatory oxidized phospholipids (OxPL) that contribute to endothelial dysfunction and plaque instability via non-platelet-dependent mechanisms (24–26). Crucially, through its structural homology with plasminogen, Lp(a) also impairs fibrinolysis, creating a systemic environment that favors thrombus persistence (13, 27, 28). In contrast, an elevated platelet count primarily reflects an increased reservoir of cellular substrate available for clot formation. Therefore, when both factors are present, they may impose a “dual hit” on the thrombotic process: Lp(a) fosters a milieu of “impaired fibrinolytic clearance,” while increased platelet count amplifies the “thrombotic formative substrate.” Because our data do not support a synergistic interaction, these effects are best understood as parallel, independent pathways whose risk contributions add to each other rather than amplifying one another. However, it is important to note that platelet count only provides information on the quantity of circulating platelets and does not directly represent their activation state or reactivity, which may be more directly related to thrombotic risk. The distinction between platelet count and platelet function is not merely a mechanistic nuance. It has direct implications for both risk prediction and therapeutic targeting. Platelet reactivity as measured by aggregometry, flow cytometry, or soluble activation markers such as P-selectin or CD40 ligand can vary considerably among individuals with similar platelet counts, and this functional heterogeneity may more accurately reflect the underlying pro-thrombotic propensity. By relying on a static platelet count as a surrogate for platelet-mediated thrombotic risk, we have likely underestimated the complexity of the biological interplay between Lp(a) and platelets. Future studies that incorporate functional platelet assays or circulating activation markers will be essential to validate the additive risk model we observed, and to clarify whether aspirin's modifying effect is more closely tied to platelet function or simply to platelet count.

Our age-stratified analysis further delineates a clear cumulative risk pattern. With advancing age, the cumulative burden of atherosclerosis increases, and vascular repair mechanisms may decline, rendering the vasculature more susceptible to acute thrombotic events when both a vulnerable plaque milieu [promoted by Lp(a)] and an enhanced thrombotic substrate (reflected by platelet count) coexist (29, 30).

From a risk management perspective, our analyses adjusted for multiple potential confounding factors, including CRP, diet, and physical activity, and found that the combined association of Lp(a) and platelet count remained independent. This indirectly supports the notion that platelet count may itself serve as a composite risk indicator integrating various influences, including genetic, inflammatory, and lifestyle factors. For individuals with genetically elevated Lp(a), whose Lp(a) levels are difficult to alter, platelet count—being variable and influenced by multiple factors—could represent a potential target for risk modulation. Prospective intervention studies are needed to verify whether modifying these adjustable factors can specifically attenuate the elevated risk associated with the combination of elevated Lp(a) and higher platelet count, thereby informing more precise preventive approaches for this high-risk population.

Finally, previous evidence regarding the cardiovascular protective effect of aspirin in individuals with high Lp(a) has been contradictory. For instance, two studies involving patient populations found that aspirin treatment significantly reduced plasma Lp(a) levels in the short term (31, 32). A retrospective study suggested that elevated plasma Lp(a) concentrations were associated with worse long-term outcomes in patients undergoing revascularization and that prolonged dual antiplatelet therapy provides benefit to these patients (33). The ASPREE trial in healthy older individuals treated with aspirin also demonstrated a reduction in ischemic events in those with genetically elevated Lp(a) levels in plasma, without an increase in bleeding events (34). However, a recent analysis integrating multiple large cohorts showed that the benefit of aspirin use did not differ by Lp(a) levels (35). These inconsistencies may stem from variations in study design (e.g., secondary vs. primary prevention), baseline population risk, or the definition of “high Lp(a).” Moreover, the clinical relevance of the interaction between Lp(a) and platelets in CVDs needs to be further defined (22).

The findings of the present study may help to reconcile these contradictory results. Aspirin use was not associated with reduced MACE risk in individuals with isolated elevated Lp(a) and normal platelet counts, whereas a trend toward potential protective effect was observed specifically in those with concomitant elevations of both biomarkers. This heterogeneity may explain the discrepant conclusions across prior studies. Positive findings in earlier reports (31, 32, 36) could be driven by a higher proportion of participants with this dual-high phenotype in their cohorts, whereas unstratified analyses of mixed populations would dilute this subgroup-specific benefit and yield null results (35). In formal interaction analyses, a significant multiplicative interaction was detected between aspirin use and platelet count, consistent with aspirin's antithrombotic mechanism of platelet inhibition and supporting platelet count as a marker to identify candidates most likely to benefit. In contrast, no interaction was observed between aspirin and Lp(a) (*P* = 0.923), in line with the concept that Lp(a) exerts its pro-atherogenic effects largely through platelet-independent pathways such as atherogenesis and impaired fibrinolysis. Given the observational design and exploratory nature of the subgroup analyses, these findings should be regarded as hypothesis-generating and require confirmation in independent cohorts.

## Study limitations

This study has several important limitations. First, the investigation is an observational epidemiological analysis based on a large biobank, and its design inherently limits causal inference. Although multivariable adjustments were performed in the statistical analyses, residual confounding due to unmeasured factors (such as prior revascularization and other subclinical inflammatory conditions) cannot be fully excluded. And, over 90% of the participants in the UK Biobank are White, which limits the generalizability of the study findings to other ethnic groups or populations from different regions. Additionally, the diagnosis of the primary endpoint (MACE) and other diseases relied on ICD-10 codes, which may be subject to misclassification. Furthermore, non-fatal stroke within the composite MACE endpoint was not stratified into ischemic and hemorrhagic subtypes. Given that aspirin reduces ischemic stroke risk but may increase hemorrhagic stroke risk, this undifferentiated endpoint could attenuate the true association between aspirin and stroke outcomes. Future studies with adjudicated stroke subtype classification are needed to clarify this relationship.

Second, the UK Biobank cohort is subject to healthy volunteer bias. Exclusion of a large number of high-risk individuals due to missing data on key variables such as Lp(a) and platelet count may have yielded a healthier analytic cohort with lower baseline risk than the source population, potentially attenuating observed associations and limiting generalizability to higher-risk groups.

Third, information on aspirin use was solely derived from a single baseline measurement based on self-report during the baseline visit. Data regarding dosage, frequency, long-term adherence, and dynamic changes in medication usage throughout the follow-up period were lacking. This time-invariant, crude classification not only prevents the exploration of dose–response relationships but also introduces misclassification bias, which may significantly underestimate or obscure the true effect of aspirin. Consequently, all conclusions related to aspirin should be regarded as hypothesis-generating rather than confirmatory. Future studies incorporating serial measurements and other antiplatelet agents are needed to clarify the dynamic relationships between these factors and cardiovascular risk. Meanwhile, residual confounding cannot be fully excluded, particularly confounding by indication and healthy user bias. Aspirin use is not randomly assigned in primary prevention; prescribing decisions are shaped by clinical judgment, baseline cardiovascular risk, frailty, comorbidity burden, concomitant medications, and physician practice patterns. Aspirin users may also differ from non-users in unmeasured factors such as subclinical disease severity, health literacy, socioeconomic status, and lifestyle habits. Although our multivariable models and IPTW sensitivity analyses adjusted for a broad range of measured covariates, these adjustments may not fully account for such differences. Furthermore, the sample size in the aspirin-user subgroup was limited. Accordingly, the observed associations between aspirin use and the risk of MACE, particularly within the subgroups, should be interpreted as non-definitive. Furthermore, in the exploratory sensitivity analysis using multiple imputation for missing covariates, the potential protective association of aspirin in the double-high subgroup was not robustly replicated, further indicating that this subgroup finding is uncertain and exploratory in nature.

Fourth, key biomarkers, including Lp(a) and platelet count, were assessed only once at baseline, thereby failing to capture their dynamic fluctuations during follow-up. Platelet count, in particular, is susceptible to a variety of acute and chronic conditions, which compromises its stability, and its static value does not directly reflect platelet function or activation status, factors more closely tied to thrombotic risk. Meanwhile, the additive interaction between the two markers did not reach statistical significance in our study, suggesting that their combined risk may approximate a simple summation of independent effects. Although the synergy index hinted at a possible synergistic trend, this indicator offers only indirect evidence. Furthermore, all interaction analyses were grounded in static biomarker quantities, without addressing the more critical dynamic functional aspects—such as platelet reactivity—which constrains a deeper understanding of the underlying biological mechanisms. Prospective studies integrating serial measurements and functional platelet assays are urgently needed to validate the biological interplay.

Fifth, although we used the Fine-Gray competing-risks model, with non-cardiovascular death treated as the competing event for cardiovascular death, this analysis addressed only a single source of competition. It did not, however, account for the interrelationships among the individual components of the composite MACE endpoint, namely, non-fatal MI, non-fatal stroke, and cardiovascular death, where the occurrence of one non-fatal event may alter the likelihood of subsequent cardiovascular death. This simplified approach may lead to overestimation of the cumulative incidence of later events, and thus the component-specific results should be interpreted with caution. Furthermore, although the proportional hazards assumption was upheld in our primary models, it remains possible that some covariates exert time-varying effects, and the current competing-risk treatment alone cannot fully capture the complex dependencies among different endpoints. Future studies that incorporate dynamic monitoring of medication use and biomarker changes, and that employ multi-state models or cause-specific hazard analyses, would be better positioned to comprehensively assess these competing relationships and to further validate our findings.

## Conclusion

Elevated Lp(a) and increased platelet count independently predict incident MACEs, with the highest risk observed in individuals with concurrent elevation of both biomarkers. Joint assessment of these two markers may refine cardiovascular risk stratification in primary prevention populations. The apparent benefit of aspirin was largely restricted to the dual-elevation subgroup and was not seen in those with isolated Lp(a) elevation. This finding provides preliminary insights for personalized preventive strategies, but remains exploratory and hypothesis-generating, warranting confirmation in well-designed prospective studies.

## Data Availability

The data analyzed in this study is subject to the following licenses/restrictions: The data used in this study are from the UK Biobank, a publicly accessible resource. However, access is not unrestricted; researchers must apply through the UK Biobank Access Management System, submit a research proposal, and obtain formal approval. After approval, data are made available under a data access agreement that prohibits redistribution and limits use to the approved project. Requests to access these datasets should be directed to data are available from the UK Biobank upon application. Researchers can apply for access via the UK Biobank Access Management System at https://ams.ukbiobank.ac.uk/ams/, following the application process detailed at https://www.ukbiobank.ac.uk/use-our-data/apply-for-access/. Access is subject to approval by the UK Biobank Access Committee and requires a signed Material Transfer Agreement. The data used in this study were obtained under UK Biobank application number 134973.
