# Supplementary material for: Lp(a), platelet count, aspirin and cardiovascular outcome: a prospective cohort analysis
Source: Front Cardiovasc Med. 2026 Sep 21;13:1932150. doi: 10.3389/fcvm.2026.1932150 (PMC13635306; doi:10.3389/fcvm.2026.1932150)
Supplement: Supplementary file 1 [file Datasheet1.docx]

**Lp(a), platelet count, aspirin and cardiovascular outcome:**

**a prospective cohort analysis**

**Supplementary Methods**

**Definitions of metabolic equivalent and diet score**

We use metabolic equivalent (MET) to quantify physical activity (PA). Based on total MET-hours per week, the population was divided into three levels: low intensity PA: <10 MET-hours/week; moderate intensity PA: 10-50 MET hours/week; high intensity PA: >50 MET-hours/week. The diet score was derived from the following dietary factors: vegetable intake ≥ 4 tablespoons/day, fruit intake ≥ 3 pieces/day, ﬁsh intake ≥ twice a week, unprocessed red meat intake ≤ twice a week, and processed meat intake ≤ twice a week. Each beneficial dietary factor received one point, with the healthy eating score ranging from 0 to 5.

**Supplement Figures**

**Supplementary** Figure 1. **Association between log-transformed Lp(a) and risk of MACE.**

**Supplementary** Figure 2. **Association between log-transformed platelet count and risk of MACE.**

**Supplementary Figure 1**. **Association between log-transformed Lp(a) and risk of MACE.**

Restricted cubic spline plot showing multivariable-adjusted hazard ratios for MACE across log-transformed Lp(a) [log (Lp(a)+0.1)]. The solid line is the point estimate, shaded area 95% CI, and blue bars represent the distribution of log-Lp(a). The reference HR=1 was set at the fitted minimum (dashed vertical line). Nonlinearity P<0.001. This Cox proportional hazards model was adjusted for age, sex, smoking status, alcohol consumption, ethnicity, diet score, body mass index, low-density lipoprotein cholesterol, triglycerides, C-reactive protein, diabetes, hypertension, atrial fibrillation, chronic kidney disease, and the use of anti-hypertensive medications, lipid-lowering agents, and other anti-platelet drugs. A constant of 0.1 was added to all Lp(a) values prior to log-transformation to handle zero or undetectable levels. Abbreviation: Lp(a), lipoprotein(a).

**Supplementary Figure 2. Association between log-transformed platelet count and risk of MACE.**

Restricted cubic spline plot for MACE across log-transformed platelet count [log (PLT+0.1)]. The solid line shows HR, shaded area 95% CI, and blue bars the distribution of log-PLT. Reference HR=1 at log (PLT)=5.50 (raw PLT≈245×10⁹/L, dashed vertical line). Nonlinearity P<0.001. Adjustment covariates are identical to those in supplementary figure 1. A constant of 0.1 was added to platelet count values prior to log-transformation to handle zero or undetectable levels. Abbreviation: PLT, platelet count.

****Supplementary Table 1. Comparison of baseline characteristics between the study participants with and without missing key variables.****

| Characteristics | Overall population  (n = 250,927) | Missing data subgroup  (n = 119,618) | P-value |
| --- | --- | --- | --- |
| Age (years) | 56.00 (49.00-62.00) | 57.00 (49.00-63.00) | <0.001 |
| Male | 115,122 (45.90a%) | 51,468 (43.00%) | <0.001 |
| Ethnicity |  |  | <0.001 |
| White | 227,830 (90.80%) | 108,391 (91.30%) |  |
| Smoking history |  |  | 0.117 |
| Yes | 148,250 (59.10%) | 69,842 (58.80%) |  |
| No | 102,677 (40.90%) | 48,918 (41.20%) |  |
| Drinking history |  |  | <0.001 |
| Yes | 241,665 (96.30%) | 113,973 (95.80%) |  |
| No | 9,262 (3.70%) | 5,046 (4.20%) |  |
| Education | 8.88 (3.51-19.11) | 9.82 (3.79-21.21) | <0.001 |
| Household income (£) |  |  | <0.001 |
| <18 000 | 41,760 (18.60%) | 21,369 (21.50%) |  |
| 18 000-30 999 | 54,545 (24.30%) | 25,148 (25.30%) |  |
| 31 000-51 999 | 61,631 (27.40%) | 26,458 (26.60%) |  |
| 52 000-100 000 | 52,090 (23.20%) | 20,849 (21.00%) |  |
| >100 000 | 14,586 (6.50%) | 5,590 (5.60%) |  |
| Physical activity |  |  | <0.001 |
| low intensity | 44,512 (17.70%) | 16,663 (13.90%) |  |
| moderate intensity | 128,100 (51.10%) | 45,858 (38.30%) |  |
| high intensity | 78,315 (31.20%) | 27,467 (23.00%) |  |
| BMI (kg/m²) | 26.39 (23.90-29.38) | 26.70 (24.09-29.93) | <0.001 |
| SBP (mmHg) | 135.00 (123.50-148.00) | 136.50 (124.50-149.50) | <0.001 |
| Height (cm) | 168.70 (162.00-176.00) | 168.00 (161.00-175.00) | <0.001 |
| Weight (kg) | 75.90 (66.00-86.80) | 76.00 (66.10-87.30) | <0.001 |
| TC (mmol/L) | 5.69 (5.00-6.43) | 5.73 (4.98-6.50) | <0.001 |
| TG (mmol/L) | 1.45 (1.02-2.10) | 1.47 (1.03-2.12) | <0.001 |
| HDL-C (mmol/L) | 1.42 (1.19-1.69) | 1.43 (1.19-1.71) | <0.001 |
| LDL-C (mmol/L) | 3.55 (3.01-4.13) | 3.56 (2.99-4.17) | <0.001 |
| CRP (mg/L) | 1.22 (0.61-2.52) | 1.31 (0.65-2.71) | <0.001 |
| Hypertension | 39,632 (15.80%) | 21,356 (17.90%) | <0.001 |
| Diabetes | 10,315 (4.10%) | 6,343 (5.30%) | <0.001 |
| **Atrial fibrillation** | 2,054 (0.80%) | 994 (0.80%) | 0.561 |
| **Chronic kidney disease** | 5,334 (2.10%) | 2,911 (2.50%) | <0.001 |
| **Use of anti-hypertensive** | 21,312 (8.50%) | 10,184 (8.60%) | 0.400 |
| **Hypoglycemic agent** | 6,456 (2.60%) | 3,939 (3.30%) | <0.001 |
| **Lipid-lowering drugs** | 18,222 (7.30%) | 9,333 (7.90%) | <0.001 |
| **Anti-platelet medications** | 24,950 (9.90%) | 12,687 (10.60%) | <0.001 |
| Diet score |  |  | <0.001 |
| 0 | 3,108 (1.20%) | 1,544 (1.30%) |  |
| 1 | 23,540 (9.40%) | 12,116 (10.10%) |  |
| 2 | 57,888 (23.10%) | 28,140 (23.50%) |  |
| 3 | 76,020 (30.30%) | 36,245(30.30%) |  |
| 4 | 63,279 (25.20%) | 29,192 (24.40%) |  |
| 5 | 27,092 (10.80%) | 12,381 (10.40%) |  |

Continuous variables with non-normal distribution are presented as median (interquartile range, IQR); categorical variables are presented as frequency (percentage). Between-group comparisons were performed using the Mann-Whitney U test for continuous variables and the chi-square test for categorical variables. Participants excluded due to missing data on Lp(a) or platelet count.

Physical activity levels were quantified using metabolic equivalent (MET) hours per week and categorized as: low intensity (< 10 MET-hours/week), moderate intensity (10-50 MET-hours/week), and high intensity (> 50 MET-hours/week).Diet score was derived from the following dietary factors: vegetable intake ≥4 tablespoons/day, fruit intake ≥3pieces/day, ﬁsh intake ≥twice a week, unprocessed red meat intake ≤twice a week, and processed meat intake ≤twice a week. Each benefical dietary factor received one point, with the healthy eating score ranging from 0 to 5.

Abbreviations: BMI, Body Mass Index; CRP, C-reactive protein; DBP, Diastolic blood pressure; HDL-C, High density lipoprotein cholesterol; LDL-C, Low density lipoprotein cholesterol; SBP, Systolic blood pressure; TC, Total cholesterol; TG, Triglycerides;

****Supplementary Table 2.**** Association of combined Lp(a) and platelet count categories with MACE risk after multiple imputation for missing covariates.

|  |  | HR (95% CI) | | |
| --- | --- | --- | --- | --- |
|  | Events/participants | Model 1 | Model 2 | Model 3 |
| Lp(a) ≤ 125 nmol/L-  platelet count ≤ 273 ×10⁹/L | 8,020/193,503 | 1 (reference) | 1 (reference) | 1 (reference) |
| Lp(a) ≤ 125 nmol/L-  platelet count > 273 ×10⁹/L | 3,389/96,791 | 1.101 (1.056-1.147) | 1.078 (1.034-1.123) | 1.074 (1.031-1.120) |
| Lp(a) > 125 nmol/L-  platelet count ≤ 273 ×10⁹/L | 1,160/23,677 | 1.229 (1.156-1.307) | 1.233 (1.159-1.311) | 1.214 (1.141-1.291) |
| Lp(a) > 125 nmol/L-  platelet count > 273 ×10⁹/L | 549/11,970 | 1.469 (1.347-1.602) | 1.438 (1.318-1.569) | 1.421 (1.303-1.551) |

Multiple imputation by chained equations (MICE) was performed to address missing data in covariates, generating 5 imputed datasets. Estimates were pooled using Rubin ‘s rules. Values are HR (95% CI) derived from Cox regression models.

Model 1 was adjusted for age, sex, smoke, drink, ethnicity, diet score, physical activity and BMI. Model 2 was adjusted as Model 1 plus LDL-c, TG and CRP. Model 3 was adjusted as Model 2 plus diabetes, hypertension, atrial fibrillation, chronic kidney disease, use of anti-hypertensive, hypoglycemic agent, lipid-lowering drugs, and other anti-platelet medications. Abbreviations: BMI, Body Mass Index; CI, Confidence interval; CRP, C-reactive protein; HR, Hazard ratio; LDL-C, Low density lipoprotein cholesterol; Lp(a), Lipoprotein(a); TG, Triglycerides;

****Supplementary Table 3.**** Inverse probability of treatment weighting (IPTW)-adjusted association of combined Lp(a) and platelet count categories with MACE risk.

|  | HR (95% CI) | | |
| --- | --- | --- | --- |
|  | Model 1 | Model 2 | Model 3 |
| Lp(a) ≤ 125 nmol/L-  platelet count ≤ 273 ×10⁹/L | 1 (reference) | 1 (reference) | 1 (reference) |
| Lp(a) ≤ 125 nmol/L-  platelet count > 273 ×10⁹/L | 0.999 (0.930-1.073) | 0.979 (0.911-1.052) | 0.987 (0.919-1.061) |
| Lp(a) > 125 nmol/L-  platelet count ≤ 273 ×10⁹/L | 1.165 (1.053-1.290) | 1.175 (1.062-1.301) | 1.152 (1.041-1.275) |
| Lp(a) > 125 nmol/L-  platelet count > 273 ×10⁹/L | 1.413 (1.205-1.657) | 1.389 (1.185-1.629) | 1.381 (1.178-1.619) |

IPTW was performed to balance measured covariates between aspirin users and non-users. Values are HR (95% CI) derived from Cox regression models.

Model 1 was adjusted for age, sex, smoke, drink, ethnicity, diet score, physical activity and BMI. Model 2 was adjusted as Model 1 plus LDL-c, TG and CRP. Model 3 was adjusted as Model 2 plus diabetes, hypertension, atrial fibrillation, chronic kidney disease, use of anti-hypertensive, hypoglycemic agent, lipid-lowering drugs, and other anti-platelet medications. Abbreviations: BMI, Body Mass Index; CI, Confidence interval; CRP, C-reactive protein; HR, Hazard ratio; LDL-C, Low density lipoprotein cholesterol; Lp(a), Lipoprotein(a); TG, Triglycerides;

Supplementary Table 4. Balance of covariates between aspirin users and non-users before and after inverse probability of treatment weighting.

| Variable | SMD Unweighted | SMD weighted |
| --- | --- | --- |
| Age | 0.567 | -0.048 |
| Sex | 0.145 | 0.026 |
| Smoke | 0.061 | 0.003 |
| Drink | -0.003 | 0.001 |
| Ethnicity | -0.009 | 0.024 |
| Diet score | 0.013 | -0.029 |
| Low-intensity physical activity | 0.020 | 0.006 |
| Moderate-intensity physical activity | -0.005 | 0.003 |
| High-intensity physical activity | -0.014 | -0.008 |
| BMI | 0.315 | 0.014 |
| LDL-C | -0.481 | 0.082 |
| TG | 0.144 | 0.040 |
| CRP | 0.069 | 0.011 |
| Diabetes | 0.091 | 0.001 |
| Hypertension | 0.162 | 0.008 |
| **Atrial fibrillation** | 0.012 | 0.001 |
| **Chronic kidney disease** | 0.030 | 0.002 |
| **Use of anti-hypertensive** | 0.234 | 0.004 |
| **Hypoglycemic agent** | 0.112 | -0.001 |
| **Lipid-lowering drugs** | 0.258 | 0.001 |
| **Other anti-platelet medications** | -0.002 | -0.001 |

Physical activity levels were quantified using metabolic equivalent (MET) hours per week and categorized as: low intensity (< 10 MET-hours/week), moderate intensity (10-50 MET-hours/week), and high intensity (> 50 MET-hours/week). Diet score was derived from the following dietary factors: vegetable intake ≥4 tablespoons/day, fruit intake ≥ 3pieces/day, ﬁsh intake ≥twice a week, unprocessed red meat intake ≤twice a week, and processed meat intake ≤twice a week. Each beneficial dietary factor received one point, with the healthy eating score ranging from 0 to 5.

Abbreviations: BMI, Body Mass Index; CRP, C-reactive protein; LDL-C, Low density lipoprotein cholesterol; SMD, standardized mean difference; TG, Triglycerides;

****Supplementary Table 5.**** Age-associated risk of Lp(a) levels and platelet count for MACE risk.

| Age group | HR (95% CI) |
| --- | --- |
| 45-65 years |  |
| Lp(a) ≤ 125 nmol/L-platelet count ≤ 273 ×10⁹/L | 1 (reference) |
| Lp(a) ≤ 125 nmol/L-platelet count > 273 ×10⁹/L | 1.043 (0.988-1.102) |
| Lp(a) > 125 nmol/L-platelet count ≤ 273 ×10⁹/L | 1.211 (1.116-1.314) |
| Lp(a) > 125 nmol/L-platelet count > 273 ×10⁹/L | 1.264 (1.120-1.427) |
| > 65 years |  |
| Lp(a) ≤ 125 nmol/L-platelet count ≤ 273 ×10⁹/L | 1 (reference) |
| Lp(a) ≤ 125 nmol/L-platelet count > 273 ×10⁹/L | 1.079 (0.973-1.198) |
| Lp(a) > 125 nmol/L-platelet count ≤ 273 ×10⁹/L | 1.236 (1.073-1.424) |
| Lp(a) > 125 nmol/L-platelet count > 273 ×10⁹/L | 1.657 (1.336-2.055) |

Values are HR (95% CI) derived from Cox regression models.

Model was adjusted for age, sex, smoke, drink, ethnicity, diet score, physical activity, BMI, LDL-c, TG, CRP, **diabetes, hypertension, atrial fibrillation, chronic kidney disease, use of anti-hypertensive, hypoglycemic agent, lipid-lowering drugs, and other anti-platelet medications.** Abbreviations: BMI, Body Mass Index; CI, Confidence interval; CRP, C-reactive protein; HR, Hazard ratio; LDL-C, Low density lipoprotein cholesterol; Lp(a), Lipoprotein(a); TG, Triglycerides;

****Supplementary Table 6****. ****Sub-distribution hazard ratios for cardiovascular death by combined categories of Lp(a) and platelet count: a competing risk analysis.****

|  | Cardiovascular death |
| --- | --- |
|  | sHR (95% CI) |
| Lp(a) ≤ 125 nmol/L-platelet count ≤ 273 ×10⁹/L | 1 (reference) |
| Lp(a) ≤ 125 nmol/L-platelet count > 273 ×10⁹/L | 1.068 (0.951-1.202) |
| Lp(a) > 125 nmol/L-platelet count ≤ 273 ×10⁹/L | 1.205 (1.011-1.439) |
| Lp(a) > 125 nmol/L-platelet count > 273 ×10⁹/L | 1.340 (1.021-1.714) |

Fine-Gray competing risk models were used to estimate sub-distribution hazard ratios (sHR) and 95% confidence intervals (CI) for cardiovascular death, with non-cardiovascular death as the competing event. All models were adjusted for age, sex, smoke, drink, ethnicity, diet score, physical activity, BMI, LDL-c, TG, CRP, **diabetes, hypertension, atrial fibrillation, chronic kidney disease, use of anti-hypertensive, hypoglycemic agent, lipid-lowering drugs, and other anti-platelet medications.** Abbreviations: BMI, Body Mass Index; CRP, C-reactive protein; Low density lipoprotein cholesterol; Lp(a), Lipoprotein(a); TG, Triglycerides.

****Supplementary Table 7.**** Cox proportional hazards model assessing the combined impact of Lp(a) levels and platelet count on MACE risk among the groups categorized based on the use of aspirin medication.

|  | NO aspirin medication  (n = 226,310) | | Aspirin medication  (n = 24,617) | |
| --- | --- | --- | --- | --- |
|  | Events/participants | HR (95% CI) | Events/participants | HR (95% CI) |
| Lp(a) ≤ 125 nmol/L-  platelet count ≤ 273 ×10⁹/L | 4,870/135,863 | 1 (reference) | 1,385/15,037 | 1 (reference) |
| Lp(a) ≤ 125 nmol/L-  platelet count > 273 ×10⁹/L | 2,036/65,863 | 1.110 (1.052-1.171) | 457/6,807 | 0.979 (0.879-1.090) |
| Lp(a) > 125 nmol/L-  platelet count ≤ 273 ×10⁹/L | 710/16,527 | 1.221 (1.128-1.321) | 211/1,901 | 1.297 (1.123-1.499) |
| Lp(a) > 125 nmol/L-  platelet count > 273 ×10⁹/L | 325/8,057 | 1.441 (1.287-1.615) | 73/872 | 1.091 (0.857-1.389) |

Values are HR (95% CI) derived from Cox regression models.

Model was adjusted for age, sex, smoke, drink, ethnicity, diet score, physical activity, BMI, LDL-c, TG, CRP, diabetes, hypertension, atrial fibrillation, chronic kidney disease, use of anti-hypertensive, hypoglycemic agent, lipid-lowering drugs, and other anti-platelet medications. Abbreviations: BMI, Body Mass Index; CI, Confidence interval; CRP, C-reactive protein; HR, Hazard ratio; LDL-C, Low density lipoprotein cholesterol; Lp(a), Lipoprotein(a); TG, Triglycerides.

****Supplementary Table 8.**** Lp(a)-associated MACE risk in the entire cohort and according to platelet count among the groups categorized based on the use of aspirin medication.

|  | NO aspirin medication  (n = 226,310) | | | Aspirin medication  (n = 24,617) | | |
| --- | --- | --- | --- | --- | --- | --- |
|  | HR (95% CI) | | | HR (95% CI) | | |
|  | Model 1 | Model 2 | Model 3 | Model 1 | Model 2 | Model 3 |
| **Entire Cohort** |  |  |  |  |  |  |
| Lp(a) ≤ 125 nmol/l | 1 (reference) | 1 (reference) | 1 (reference) | 1 (reference) | 1 (reference) | 1 (reference) |
| Lp(a) >125 nmol/l | 1.272  (1.191-1.358) | 1.250  (1.170-1.334) | 1.224  (1.165-1.329) | 1.235  (1.090-1.399) | 1.255  (1.107-1.422) | 1.246  (1.100-1.413) |
| platelet count ≤ 273 ×10⁹/L | 1 (reference) | 1 (reference) | 1 (reference) | 1 (reference) | 1 (reference) | 1 (reference) |
| platelet count > 273 ×10⁹/L | 1.131  (1.076-1.188) | 1.090  (1.038-1.146) | 1.097  (1.044-1.153) | 0.933  (0.843-1.031) | 0.930  (0.841-1.029) | 0.929  (0.840-1.028) |
| **With low**  **platelet count** |  |  |  |  |  |  |
| Lp(a) ≤ 125 nmol/l | 1 (reference) | 1 (reference) | 1 (reference) | 1 (reference) | 1 (reference) | 1 (reference) |
| Lp(a) > 125 nmol/l | 1.249  (1.154-1.351) | 1.228  (1.134-1.328) | 1.217  (1.124-1.317) | 1.213  (1.049-1.402) | 1.234  (1.067-1.427) | 1.224  (1.058-1.415) |
| **With elevated platelet count** |  |  |  |  |  |  |
| Lp(a) ≤ 125 nmol/l | 1 (reference) | 1 (reference) | 1 (reference) | 1 (reference) | 1 (reference) | 1 (reference) |
| Lp(a) > 125 nmol/l | 1.324  (1.178-1.489) | 1.300  (1.156-1.462) | 1.309  (1.164-1.473) | 1.295  (1.011-1.659) | 1.313  (1.025-1.682) | 1.310  (1.022-1.679) |

Values are HR (95% CI) derived from Cox regression models.

Model 1 was adjusted for age, sex, smoke, drink, ethnicity, diet score, physical activity and BMI. Model 2 was adjusted as Model 1 plus LDL-c, TG and CRP. Model 3 was adjusted as Model 2 plus **diabetes, hypertension, atrial fibrillation, chronic kidney disease, use of anti-hypertensive, hypoglycemic agent, lipid-lowering drugs, and other anti-platelet medications.** Abbreviations: BMI, Body Mass Index; CI, Confidence interval; CRP, C-reactive protein; HR, Hazard ratio; LDL-C, Low density lipoprotein cholesterol; Lp(a), Lipoprotein(a); TG, Triglycerides.

****Supplementary Table 9.**** ****Hazard ratios for multiplicative interaction terms between aspirin, Lp(a), and platelet count on MACE.****

|  | Unadjusted model | | Fully adjusted model | |
| --- | --- | --- | --- | --- |
|  | HR (95% CI) | P-value | HR (95% CI) | P-value |
| Lp(a)*PLT | 1.076 (0.948-1.221) | 0.255 | 1.071 (0.944-1.215) | 0.289 |
| Aspirin*Lp(a) | 0.994 (0.864-1.145) | 0.938 | 0.993 (0.862-1.143) | 0.923 |
| Aspirin*PLT | 0.834 (0.747-0.930) | 0.001 | 0.874 (0.783-0.975) | 0.016 |

Cox proportional hazards models were used to assess multiplicative interaction effects. Interaction terms are denoted by “*”. Data are presented as HR with 95% CI and the P-value for the interaction term. The “Unadjusted model” includes only the main effects of aspirin use, Lp(a), platelet count, and their respective interaction terms. The “Fully-adjusted model” further adjusts for age, sex, smoke, drink, ethnicity, diet score, physical activity, BMI, LDL-c, TG, CRP, **diabetes, hypertension, atrial fibrillation, chronic kidney disease, use of anti-hypertensive, hypoglycemic agent, lipid-lowering drugs, and other anti-platelet medications.** Abbreviations: BMI, Body Mass Index; CI, Confidence interval; CRP, C-reactive protein; HR, Hazard ratio; LDL-C, Low density lipoprotein cholesterol; Lp(a), Lipoprotein(a); PLT, Platelet Count; TG, Triglycerides.

****Supplementary Table 10****. Cox proportional hazards model assessing the combined impact of Lp(a) levels and platelet count on MACE risk among the groups categorized based on the use of aspirin medication.

|  | Overall population  (n = 250,927) | NO aspirin  medication  (n = 226,310) | Aspirin  medication  (n = 24,617) |
| --- | --- | --- | --- |
|  | HR (95% CI) | HR (95% CI) | HR (95% CI) |
| Lp(a) ≤ 60.58 nmol/L -platelet count ≤ 273 ×10⁹/L | 1 (reference) | 1 (reference) | 1 (reference) |
| Lp(a) ≤ 60.58 nmol/L -platelet count > 273 ×10⁹/L | 1.058 (1.005-1.115) | 1.093 (1.031-1.159) | 0.902 (0.801-1.015) |
| Lp(a) > 60.58 nmol/L -platelet count ≤ 273 ×10⁹/L | 1.219 (1.158-1.284) | 1.235 (1.165-1.308) | 1.156 (1.036-1.291) |
| Lp(a) > 60.58 nmol/L -platelet count > 273 ×10⁹/L | 1.348 (1.251-1.452) | 1.376 (1.267-1.494) | 1.174 (0.989-1.393) |

Values are HR (95% CI) derived from Cox regression models.

Model was adjusted for age, sex, smoke, drink, ethnicity, diet score, physical activity, BMI, LDL-c, TG, CRP, **diabetes, hypertension, atrial fibrillation, chronic kidney disease, use of anti-hypertensive, hypoglycemic agent, lipid-lowering drugs, and other anti-platelet medications.** Abbreviations: BMI, Body Mass Index; CI, Confidence interval; CRP, C-reactive protein; HR, Hazard ratio; LDL-C, Low density lipoprotein cholesterol; Lp(a), Lipoprotein(a); TG, Triglycerides.

****Supplementary Table 11****. Cox proportional hazards model assessing the combined impact of Lp(a) levels and platelet count on MACE risk among the groups categorized based on the use of aspirin medication.

|  | Overall population  (n = 250,927) | NO aspirin  medication  (n = 226,310) | Aspirin  medication  (n = 24,617) |
| --- | --- | --- | --- |
|  | HR (95% CI) | HR (95% CI) | HR (95% CI) |
| Lp(a) ≤ 20 nmol/L-platelet count ≤ 245 ×10⁹/L | 1 (reference) | 1 (reference) | 1 (reference) |
| Lp(a) ≤ 20 nmol/L-platelet count > 245 ×10⁹/L | 1.057 (0.997-1.120) | 1.063 (0.995-1.135) | 1.011 (0.891-1.146) |
| Lp(a) > 20 nmol/L-platelet count ≤ 245 ×10⁹/L | 1.187 (1.125-1.254) | 1.186 (1.115-1.262) | 1.176 (1.050-1.317) |
| Lp(a) > 20 nmol/L-platelet count > 245 ×10⁹/L | 1.208 (1.141-1.278) | 1.225 (1.149-1.305) | 1.095 (0.967-1.240) |

Values are HR (95% CI) derived from Cox regression models.

Model was adjusted for age, sex, smoke, drink, ethnicity, diet score, physical activity, BMI, LDL-c, TG, CRP, **diabetes, hypertension, atrial fibrillation, chronic kidney disease, use of anti-hypertensive, hypoglycemic agent, lipid-lowering drugs, and other anti-platelet medications.** Abbreviations: BMI, Body Mass Index; CI, Confidence interval; CRP, C-reactive protein; HR, Hazard ratio; LDL-C, Low density lipoprotein cholesterol; Lp(a), Lipoprotein(a); TG, Triglycerides.

****Supplementary Table 12****. UK Biobank Field Codes used to ascertain main diseases.

Non-Fatal MI defined as the following ICD-10 codes

I20.x, I21.x, I22.x, I23.x, I24.x, 125.x

Non-Fatal Stroke as the following ICD-10 codes

I60.x, I61.x, I63.x, I64

Any Cardiovascular death defined as the following ICD-10 codes

I20.X, I21.X, I22.X, I23.X, I24.X, I25.X, in addition to death due to any of the codes listed for non-Fatal MI or stroke.

Diabetes as the following ICD-10 codes

E10.x, E11.x, E12.x, E13.x, E14.x

**Hypertension** as the following ICD-10 codes

I10, I11.x, I12.x, I13.x, I14.x, I15.x

Atrial fibrillation as the following ICD-10 codes

I480, I481, I482, I483

Chronic kidney disease as the following ICD-10 codes

E102, E112, I12.x, I13.x, N18.x, T861, Z940

Cerebrovascular disease as the following ICD-10 codes

I20, I21, I22, I23, I24., I25, I46, I60, I61, I63, I64.

****Supplementary Table 13****. Numerical code of ****anti-platelet medications**** in UKB.

| UKB Code | UKB Drug Category |
| --- | --- |
| 1140856214 | solprin 300mg dispersible tablet |
| 1140856224 | palaprin forte 600mg tablet |
| 1140856310 | aspergum 227mg chewing gum |
| 1140861776 | antiplatelet drug |
| 1140861778 | dipyridamole |
| 1140861780 | persantin 25mg tablet |
| 1140861800 | platet 100mg effervescent tablet |
| 1140861804 | angettes 75mg tablet |
| 1140861806 | aspirin 75mg tablet |
| 1140861832 | disprin cv 100mg m/r tablet |
| 1140864860 | nu-seals aspirin 75mg e/c tablet |
| 1140868226 | aspirin |
| 1141151924 | enprin 75mg e/c tablet |
| 1141163324 | ticlopidine |
| 1141163328 | ticlid 250mg tablet |
| 1141164044 | isosorbide mononitrate+aspirin |
| 1141167844 | dipyridamole+aspirin |
| 1141167848 | asasantin retard m/r capsule |
| 1141168318 | clopidogrel |
| 1141168322 | plavix 75mg tablet |
| 1141167844 | dipyridamole+aspirin |
| 1141177826 | Micropirin 75mg e/c tablet |
| 1141181150 | cilostazol |
| 1141181154 | Pletal 100mg tablet |

****Supplementary Table 14****. UK Biobank medication codes used to define ****hypoglycemic agent.****

| UKB Code | UKB Drug Category |
| --- | --- |
| 1140883066 | insulin product |
| 1140884600 | metformin |
| 1140874686 | glucophage 500mg tablet |
| 1140857494 | glibornuride |
| 1140857496 | glutril 25mg tablet |
| 1140857500 | glymidine |
| 1140857502 | gondafon 500mg tablet |
| 1140857508 | glucotard 5g/sachet mini-tablet |
| 1140857586 | dimelor 500mg tablet |
| 1140857584 | acetohexamide |
| 1140868902 | acarbose |
| 1140868908 | glucobay 50mg tablet |
| 1140874646 | glipizide |
| 1140874650 | glibenese 5mg tablet |
| 1140874652 | minodiab 2.5mg tablet |
| 1140874658 | gliquidone |
| 1140874664 | tolazamide |
| 1140874674 | tolbutamide |
| 1140874678 | glyconon 500mg tablet |
| 1140874706 | chlorpropamide |
| 1140874718 | glibenclamide |
| 1140874720 | glibenclamide + metformin |
| 1140874728 | euglucon 2.5mg tablet |
| 1140874736 | diabetamide 2.5mg tablet |
| 1140874744 | gliclazide |
| 1140874746 | diamicron 80mg tablet |
| 1141152590 | glimepiride |
| 1141157284 | glipizide product |
| 1141168660 | repaglinide |
| 1141168668 | novonorm 0.5mg tablet |
| 1141171646 | pioglitazone |
| 1141171652 | actos 15mg tablet |
| 1141173882 | nateglinide |
| 1141177600 | rosiglitazone |
| 1141177606 | avandia 4mg tablet |
| 1141189090 | rosiglitazone 1mg / metformin 500mg tablet |
| 1141189094 | avandamet 1mg / 500mg tablet |
